# MacroH2A1 Regulation of Poly(ADP-Ribose) Synthesis and Stability Prevents Necrosis and Promotes DNA Repair

**DOI:** 10.1128/MCB.00230-19

**Published:** 2019-12-11

**Authors:** Penelope D. Ruiz, Gregory A. Hamilton, Jong Woo Park, Matthew J. Gamble

**Affiliations:** a Department of Molecular Pharmacology, Albert Einstein College of Medicine, Bronx, New York, USA; b Department of Cell Biology, Albert Einstein College of Medicine, Bronx, New York, USA

**Keywords:** DNA damage, PARP, chromatin, macroH2A1, necrosis

## Abstract

Through its ability to bind the ends of poly(ADP-ribose) (PAR) chains, the function of the histone variant macroH2A1.1, including its ability to regulate transcription, is coupled to PAR polymerases (PARPs). PARP1 also has a major role in DNA damage response (DDR) signaling, and our results show that macroH2A1 alters the kinetics of PAR accumulation following acute DNA damage by both suppressing PARP activity and simultaneously protecting PAR chains from degradation. In this way, we demonstrate that macroH2A1 prevents cellular NAD^+^ depletion, subsequently preventing necrotic cell death that would otherwise occur due to PARP overactivation. We also show that macroH2A1-dependent PAR stabilization promotes efficient repair of oxidative DNA damage. While the role of PAR in recruiting and regulating macrodomain-containing proteins has been established, our results demonstrate that, conversely, macrodomain-containing proteins, and specifically those containing macroH2A1, can regulate PARP1 function through a novel mechanism that promotes both survival and efficient repair during DNA damage response.

## Abstract (precis)

Through its ability to bind the ends of poly(ADP-ribose) (PAR) chains, the function of the histone variant macroH2A1.1, including its ability to regulate transcription, is coupled to PAR polymerases (PARPs). PARP1 also has a major role in DNA damage response (DDR) signaling, and our results show that macroH2A1 alters the kinetics of PAR accumulation following acute DNA damage by both suppressing PARP activity and simultaneously protecting PAR chains from degradation.

## INTRODUCTION

Poly(ADP-ribosyl)ation (PARylation), the NAD^+^-dependent addition of poly(ADP-ribose) (PAR) chains on target proteins, is a posttranslation modification that plays important roles in the regulation of transcription, DNA repair, and cell death (1–3). PAR formation is catalyzed by a family of PAR polymerases (PARPs), of which PARP1 is the most active and well-studied member. PARP1’s functions in DNA damage responses (DDRs) are mediated by its regulated synthesis of PAR, using NAD^+^ as a donor of ADP-ribose units. PARP1 is allosterically activated upon binding to DNA single- and double-strand breaks (DSBs) generated by genotoxic stress, such as oxidative DNA damage (4), and this functions as a rapid cellular sensor for detecting DNA damage. The PAR chains produced by activated PARP1 promote DNA repair by recruiting DNA repair factors to the sites of DNA damage (5), which are then also frequently PARylated (6). Furthermore, PAR chains have roles in multiple repair pathways, including both long- and short-patch base excision repair (BER), single-strand break repair (SSBR), DSB repair, and the removal of bulky adducts (7). In this way, PARP1 can promote cellular survival during a DNA damage response.

However, not all PARP1 activity promotes cell survival; under conditions of excessive DNA damage, PARP1 can also promote cell death by several mechanisms. For example, translocation of protein-free PAR chains from the nucleus to mitochondria triggers parthanatos, a process in which apoptosis-inducing factor (AIF) is released from the mitochondria in response to PAR, translocates to the nucleus, and initiates a form of caspase-independent apoptosis (8). In a second mechanism, PARP1 can be “overactivated” in response to high levels of DNA damage, which can lead to the depletion of its critical substrate NAD^+^, which in turn leads to loss of ATP, resulting in necrosis (8).

The amount of PAR that accumulates during a DNA damage response is a function of the amount and type of damage, the rate of PAR synthesis by PARPs, and the rate of PAR turnover. Protein-conjugated PAR can be hydrolyzed to free PAR and/or monomeric ADP-ribose by either ADP-ribosylhydrolase (ARH3) or PAR glycohydrolase (PARG), which possess both endoglycosidic and exoglycosidic cleavage activities (9–11). While PAR synthesis is highly regulated, the enzymatic activities of PARG and ARH3 are for the most part ubiquitously expressed and constitutively active (12).

Macrodomains are roughly 25-kDa conserved globular domains that typically harbor the ability to interact with monomeric ADP-ribose and/or PAR chains (13, 14). The functions of macrodomain-containing proteins are regulated by interaction with PAR (15–17). For example, the macrodomain-containing protein and chromatin-remodeling enzyme ALC1 is recruited to sites of DNA damage by interacting with PAR chains (15, 17). In addition, the ability of ALC1 to remodel nucleosomes requires its interaction with PAR (15).

The macrodomain-containing histone variant macroH2A1 comes in two alternatively spliced forms, macroH2A1.1 and macroH2A1.2, the former of which can interact with PAR chains (18, 19). While normal cells typically express both macroH2A1 variants, macroH2A1.1 expression is specifically lost in many cancers and functions as a tumor suppressor (20–24). Similar to other macrodomain-containing factors, PAR binding regulates the functions of macroH2A1.1, including repression of cellular proliferation, repression of metastatic potential, regulation of histone posttranslational modifications, and regulation of gene expression (19, 20, 22, 23, 25–28). Recent evidence suggests that macroH2A1 is also involved in DNA repair. Separate reports indicate that macroH2A1.1 and macroH2A1.2 are both recruited to DNA double-strand breaks and sites of replication stress, where macroH2A1 represses nonhomologous end joining and promotes homology-directed repair (29–31).

Here, we demonstrate that macroH2A1 promotes cell survival following oxidative DNA damage through a novel mechanism of globally modulating PAR metabolism. MacroH2A1 alters the global kinetics of PAR accumulation during a DNA damage response by both suppressing PARP activity and simultaneously suppressing the rate of PAR turnover. In this way, macroH2A1.1 prevents cellular NAD^+^ depletion and necrotic cell death that would otherwise occur due to PARP overactivation. While the role of PAR in recruiting and regulating macrodomain-containing proteins has been established, our results demonstrate that macroH2A1 promotes PAR stability while at the same time dampening PAR synthesis. The influence of macroH2A1 on PAR metabolism has two key consequences: (i) macroH2A1 suppresses necrotic cell death induced by acute oxidative stress and (ii) macroH2A1 promotes efficient repair through stabilized PAR chains. Given the current level of investigation into the utility of PARP inhibitors in cancer therapy (32–34), we believe these results have important implications for determining in which patient populations PARP inhibitors may prove most efficacious.

## RESULTS

### MacroH2A1 protects against DNA damage-induced, PARP1-mediated necrosis.

**FIG 1:**
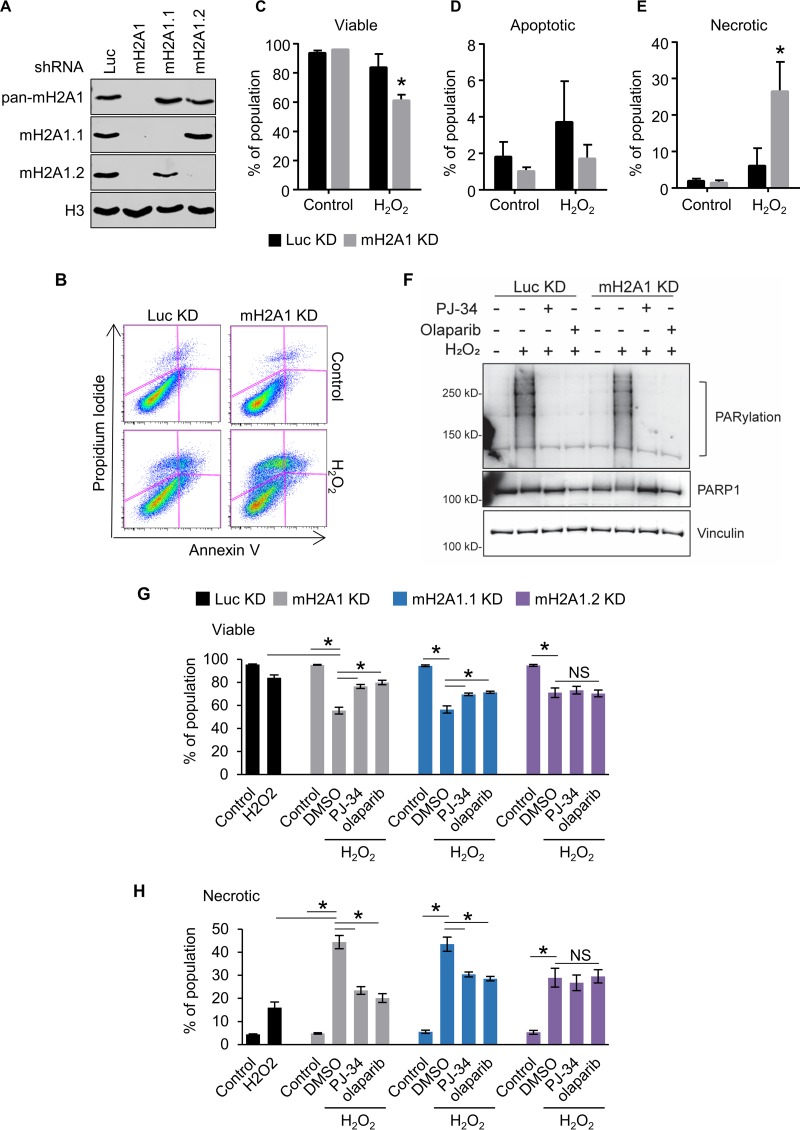
MacroH2A1 protects against DNA damage-induced necrosis. (A) Immunoblots of acid-extracted histones for macroH2A1.1 (mH2A1.1), macroH2A1.2 (mH2A1.2), and H3 as a loading control in IMR90 cells expressing shRNA directed against either luciferase (Luc KD) as a control, both macroH2A1 isoforms (mH2A1 KD), macroH2A1.1 (mH2A1.1), or macroH2A1.2 (mH2A1.2). (B) Scatterplots depicting representative propidium iodide and annexin-V costaining flow cytometry analysis of IMR90 cells expressing shRNA against Luc or mH2A1 8 h following treatment with or without H_2_O_2_ (200 μM) for 90 min. (C, D, and E) Percentages of viable (C), apoptotic (D), and necrotic (E) cells. (F) Immunoblots of whole-cell extracts from IMR90 cells expressing shRNA against Luc or mH2A1 following treatment with 125 μM H_2_O_2_ for 15 min. Where indicated, the cells were pretreated with 10 μM PJ-34 or 1 μM olaparib. (G and H) Histograms showing percentages of viable (G) and necrotic (H) cells as measured by fluorescence-based CytoTox-Glo cytotoxicity assay 6 h after treatment. Where indicated, the cells were treated for 90 min with 200 μM H_2_O_2_ with or without 30 min pretreatment with 10 μM PJ-34 or 1 μM olaparib. *, *P* < 0.05; NS, not significant; Student's *t* test. The bars and error bars represent the means ± SEM of the results of three independent experiments.

To examine the role of macroH2A1 in the DNA damage response, we used short hairpin RNA (shRNA) to simultaneously deplete IMR90 primary lung fibroblasts of both macroH2A1.1 and macroH2A1.2 or to specifically deplete them of either macroH2A1.1 or macroH2A1.2 (Fig. 1A). Control and macroH2A1-depleted cells were treated with the DNA-damaging agent hydrogen peroxide (H_2_O_2_) for 90 min, moved into normal medium, and incubated for 8 h, followed by annexin V and propidium iodide (PI) staining (Fig. 1B to E). Early apoptotic cells are positive for annexin V and negative for PI, while early necrotic cells are PI positive and annexin V negative. Even at this early time point, we could detect a significant decrease in cell viability in macroH2A1-depleted cells compared to controls (Fig. 1C). While no significant difference in the level of early apoptotic cells was observed (Fig. 1D), a greater than 4-fold increase in necrotic cells was found when macroH2A1 was depleted (Fig. 1E). This increased necrosis in macroH2A1-depleted cells was confirmed using the CytoTox-Glo cytotoxicity assay, a fluorescence measurement of a protease released by necrotic cells (Fig. 1G and H). Interestingly, cells expressing either shRNA against luciferase (Luc) or mH2A1.2 have significantly increased cell viability following hydrogen peroxide treatment compared to cells knocked down for total macroH2A1 (macroH2A1 KD cells) or macroH2A1.1 (Fig. 1G and H).

MacroH2A1.1 differs from its splice variant, macroH2A1.2, in its ability to interact with PAR and PARP1 (35). The macrodomain of macroH2A1.1 specifically binds to the ends of PAR chains (35). Although they differ with respect to the roles of the splice variants, two reports suggest that macroH2A1 can repress PARP1 enzymatic activity *in vitro* (36, 37). Given the ability of macroH2A1.1 to interact with PAR, we evaluated the role of PARP activity in mediating the increased necrosis in response to oxidative DNA damage in the macroH2A1-depleted cells. The various sizes and configurations of PAR polymers make PARylation appear as a smear on Western blots. Pretreatment with either the PARP inhibitor PJ-34 or olaparib resulted in dramatically reduced PARylation (Fig. 1F). Interestingly, pretreatment with either PARP inhibitor suppressed the increased sensitivity to DNA damage caused by depletion of macroH2A1.1 but had no effect on cells that still expressed macroH2A1.1, such as the macroH2A1.2-depleted cells (Fig. 1G and H). The ability of PARP inhibition to suppress the effect of macroH2A1 depletion, and specifically macroH2A1.1 depletion, on DNA damage sensitivity suggests that macroH2A1.1 represses PARP-mediated necrosis following H_2_O_2_ treatment.

### MacroH2A1 prevents PARP1-mediated NAD^+^ depletion upon DNA damage.

**FIG 2:**
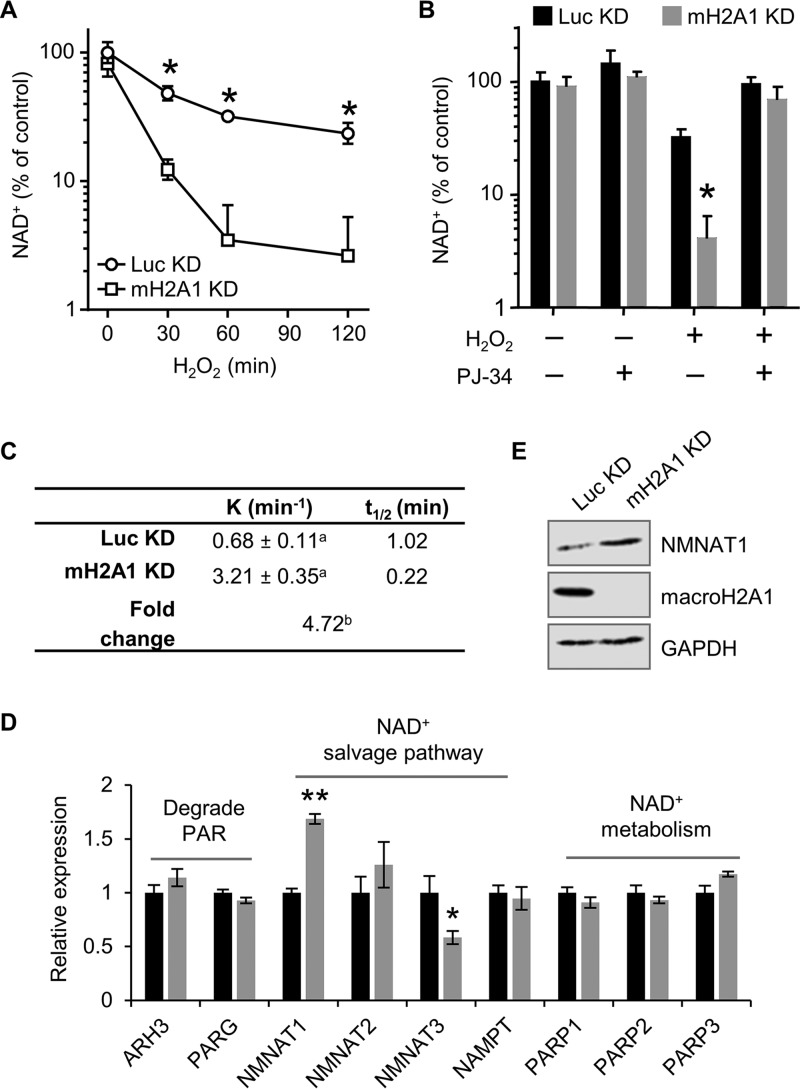
MacroH2A1 prevents NAD^+^ depletion upon DNA damage. (A) Relative cellular NAD^+^ levels in IMR90 cells expressing shRNA against macroH2A1 (mH2A1 KD) or luciferase (Luc KD) as a control following 125 μM H_2_O_2_ treatment for the indicated times. (B) NAD^+^ levels relative to control for mH2A1 KD and Luc KD IMR90 cells treated for 2 h with 125 μM H_2_O_2_ and 10 μM PJ-34 where indicated. The bars and error bars represent the means and SEM of the results of at least three independent experiments. *, *P* < 0.05; Student's *t* test. (C) Rate constant (*K*) and half-life (*t*_1/2_) of NAD^+^ in response to 125 μM H_2_O_2_ in control (Luc KD) and macroH2A1-depleted (mH2A1 KD) cells. a, standard error of the rate constant; b, *P* < 0.0001 (F test). (D) Relative expression (RT-PCR) of enzymes involved in NAD^+^ synthesis and metabolism in Luc KD and mH2A1 KD cells for four biological replicates. The bars and error bars represent means ± SEM. *, *P* = 0.02; **, *P* = 0.0007; Student's *t* test. (E) Immunoblots of total cell lysates for NMNAT1, macroH2A1, and GAPDH from Luc KD and mH2A1 KD cells.

The data presented above demonstrate that macroH2A1 prevents necrosis following DNA damage. Under conditions of excessive PAR synthesis, a state known as PARP overactivation, increased PARP activity can lead to depletion of cellular NAD^+^, which consequently leads to necrotic cell death (38–40). To test this, we monitored NAD^+^ levels from control and macroH2A1-depleted cells treated with H_2_O_2_ (Fig. 2A). Importantly, depletion of macroH2A1 does not result in a significant change in NAD^+^ levels under steady-state conditions, consistent with a previous report (41). MacroH2A1-depleted cells show a rapid decrease of cellular NAD^+^ levels upon H_2_O_2_ treatment compared with control cells. Experiments in which the cells were pretreated with the PARP inhibitor PJ-34 demonstrated that the increased NAD^+^ depletion observed in macroH2A1 KD cells was dependent on PARP activity (Fig. 2B).

One possible explanation for the rapid depletion of NAD^+^ in the macroH2A1 KD cells is altered expression of the enzymes involved in NAD^+^ biosynthesis and/or PAR metabolism. To test this, we performed reverse transcription-quantitative PCR (RT-qPCR) to examine the relative expression of a subset of these enzymes. We found a significant increase in NMNAT1, the limiting enzyme responsible for converting nicotinamide mononucleotide to NAD^+^ in the nucleus (Fig. 2D and E). Given that the majority of NAD^+^ usage during a DNA damage response occurs in the nucleus from PARP1 activity (42), these expression changes cannot explain the rapid loss of NAD^+^ during the response. Together, our results demonstrate that macroH2A1 can lower the rate of cellular NAD^+^ consumption by preventing PARP overactivation.

### MacroH2A1 regulates the kinetics of PARP activity upon DNA damage.

Allosteric activation of PARP1 enzymatic activity and the corresponding accumulation of PAR chains are hallmarks of the DNA damage response. MacroH2A1 binds to the ends of PAR chains (14), thereby blocking the linear site preferred for further polymerization. We hypothesized that by binding to the ends of PAR chains, the macrodomain of macroH2A1.1 may inhibit global PARP activity and thereby preserve cellular NAD^+^ during the DNA damage response. This model predicted that in the absence of macroH2A1.1 we should observe increased levels of the product of NAD^+^ consumption by PARP1, namely, PAR chains.

**FIG 3:**
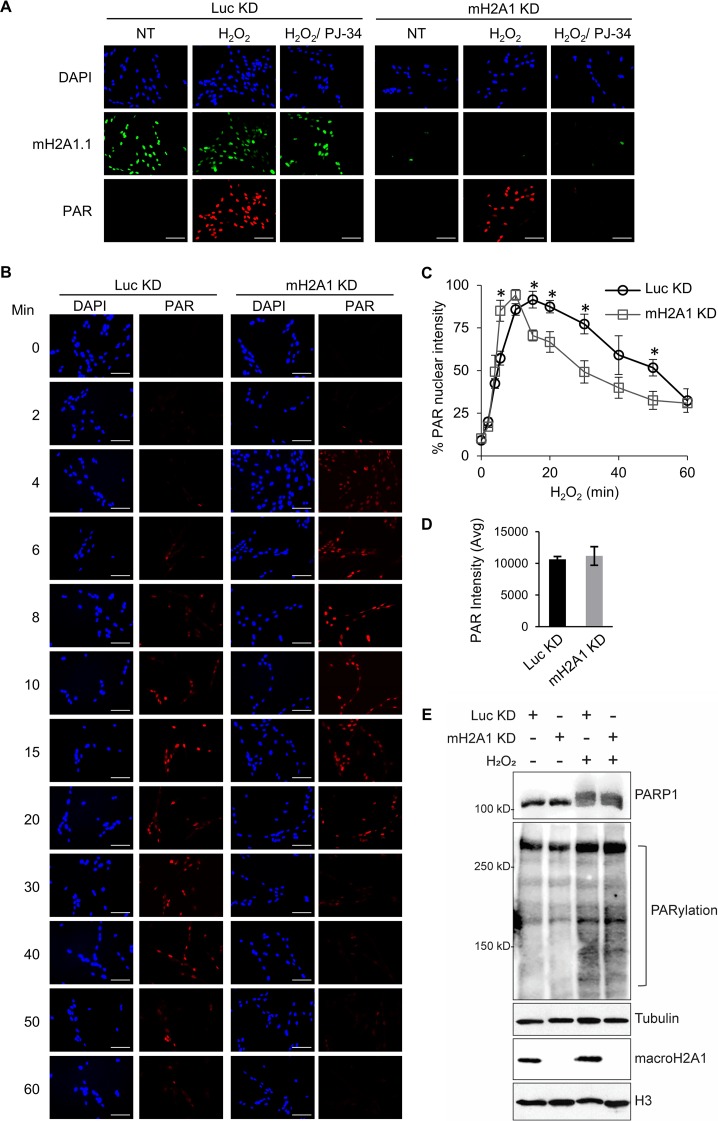
MacroH2A1 alters the kinetics of PAR accumulation upon oxidative DNA damage. (A) Immunofluorescence for PAR and macroH2A1 with DAPI counterstaining of IMR90 cells expressing shRNA against macroH2A1 (mH2A1 KD) or luciferase (Luc KD) as a control following treatment with 125 μM H_2_O_2_ for 15 min. Where indicated, the cells were pretreated with 10 μM PJ-34. Loss of PAR signal in the PJ-34-treated samples indicates that the PAR antibody is highly specific for PAR chains. (B) Immunofluorescence for PAR with DAPI counterstaining in mH2A1 KD or Luc KD IMR90 cells following treatment with 125 μM H_2_O_2_ for the indicated times. Scale bars, 100 μm. (C) PAR stability assay described in the legend to panel B for mH2A1 KD or Luc KD IMR90 cells. (D) Average total peak PAR intensities for the experiment described in the legend to panel B. (E) Immunoblots for total cell lysates and acid-extracted histones in control (Luc KD) or macroH2A1-depleted (mH2A1 KD) IMR90 cells for the indicated antibodies. Luc KD cells were treated with H_2_O_2_ for 10 min, whereas mH2A1 KD cells were treated for 15 min. (C and D) Means ± SEM of the results of three independent experiments are shown. *, *P* < 0.05; Student's *t* test.

We sought to monitor PAR by immunofluorescence to test the effect of macroH2A1 on PARP1 functional output. We confirmed the specificity of the PAR antibody by demonstrating that the signal observed in immunofluorescence was dependent on both the presence of a DNA-damaging agent and PARP activity (Fig. 3A). Using this assay, we then examined the kinetics of PAR accumulation following DNA damage in macroH2A1-depleted cells or control cells (Fig. 3B and C). The rate of PAR accumulation is somewhat higher in macroH2A1-depleted cells (maximum at 10 min) than in control cells (maximum at 15 min) treated with H_2_O_2_ (Fig. 3C). Overall, the two cell lines displayed the same amount of PAR at the peak of accumulation as measured by PAR immunofluorescence and immunoblotting (Fig. 3D and F). In addition, while the control cells largely maintained the elevated level of PAR at 30 min, the level of PAR in macroH2A1-depleted cells was reduced to 50% of maximal levels. Overall, PAR levels were more transient in the macroH2A1-depleted cells, while elevated PAR levels were better maintained in control cells. Together, these results suggest that macroH2A1 regulates the metabolism of PAR chains during a DNA damage response by promoting prolonged accumulation of PAR.

### MacroH2A1 enhances the stability of PAR chains.

The data presented above demonstrated that macroH2A1 can regulate the kinetics of PAR signaling during DNA damage response. However, the data also presented an apparent paradox. Upon treatment with H_2_O_2_, we observed rapid depletion of NAD^+^, which could be prevented with PARP inhibitors (Fig. 2A to C), and so we expected to see a corresponding increase in the total amount of PAR generated. However, the peak levels of PAR found following the DDR were similar in the two lines (Fig. 3D and F). The macroH2A1-depleted cells exhibited only subtle changes in total PAR accumulation kinetics, which did not explain the dramatic PARP1-dependent loss of NAD^+^ following acute DNA damage. However, an increased rate of PAR turnover, or degradation, could explain this apparent paradox. We hypothesized that macroH2A1-depleted cells use more NAD^+^ to maintain an equivalent level of PAR chains because PAR itself is degraded at a higher rate in the absence of macroH2A1.1 capping the ends of the chains.

**FIG 4:**
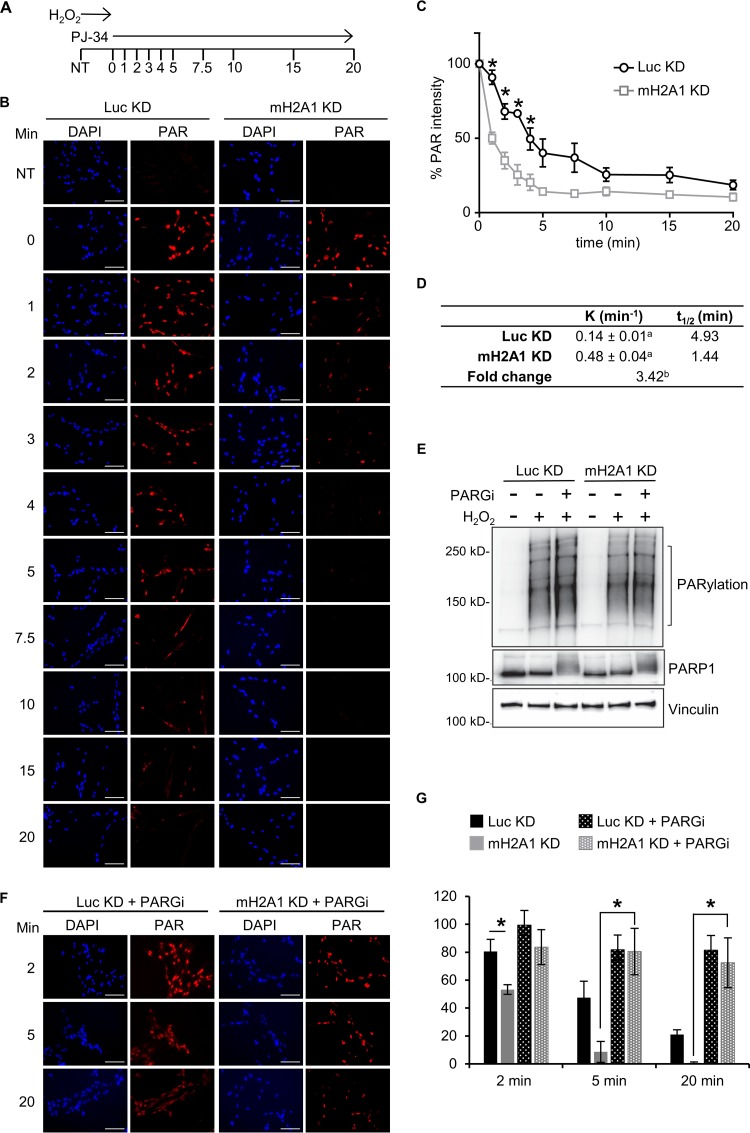
MacroH2A1 regulates PAR stability. (A) Schematic of PAR stability experiment. Cells were treated with H_2_O_2_ for 12 min to allow peak levels of PAR to accumulate; 10 μM PJ-34 was then added to prevent further PAR synthesis. PAR levels were monitored over the indicated time points (minutes). NT, not treated with H_2_O_2_. (B) Representative immunofluorescence images for the PAR stability assay described in the legend to panel A for IMR90 cells expressing shRNA against macroH2A1 (mH2A1 KD) or luciferase (Luc KD) as a control. Scale bars, 100 μm. (C) Average relative intensities of PAR staining for three independent experiments as described in the legend to panel A. The symbols and error bars represent means ± SEM. *, *P* < 0.05; Student's *t* test. (D) Rate constant (*K*) of PAR degradation and half-life (*t*_1/2_) of PAR in control (Luc KD) and macroH2A1-depleted (mH2A1 KD) cells. a, standard error of the rate constant; b, *P* < 0.0001 (F test). (E) Immunoblots for total cell lysates in control (Luc KD) or macroH2A1-depleted (mH2A1 KD) IMR90 cells for the indicated antibodies. The cells were treated with H_2_O_2_ for 12 min, in addition to 0.1 μM PARG inhibitor (PARGi) PDD00017273 where indicated. (F) Representative immunofluorescence images for the PAR stability assay described in the legend to panel A for control (Luc KD) and mH2A1-depleted (mH2A1 KD) IMR90 cells treated with 0.1 μM PARGi. (G) Average relative intensities of PAR staining for three independent experiments as described in the legend to panel F. The bars and error bars represent means ± SEM. *, *P* < 0.05; Student's *t* test.

PAR chains can be degraded by the actions of glycohydrolases, such as PARG and ARH3. Recent studies have demonstrated that the PARG structure is reminiscent of a macrodomain (9). A key feature of many macrodomains is their ability to bind to the ends of PAR chains. Recent data suggest that PARG functions primarily as an exoglycohydrolase, digesting the chains from their free ends (9). Together, these facts led us to hypothesize that macroH2A1.1 may repress PARG activity by competitively binding to PARG’s favored substrate, the ends of PAR chains. To test this hypothesis, we examined the rates of PAR degradation in control and macroH2A1-depleted cells using PAR immunofluorescence. Following treatment of control and macroH2A1-depleted cells with H_2_O_2_ for 12 min to allow the accumulation of peak levels of PAR chains, the PARP inhibitor PJ-34 was added to prevent further PAR synthesis, and the rate of loss of PAR immunofluorescence was monitored (Fig. 4). In control cells, our results indicated that PAR chains have a half-life of roughly 5 min, equivalent to the half-life observed in other studies (43). Strikingly, in macroH2A1-depleted cells, there was a 3.4-fold decrease in the PAR half-life (Fig. 4D). Furthermore, inhibition of PARG activity with the potent and selective PDD00017273 compound (44) significantly stabilized PAR chains in macroH2A1-depleted cells (Fig. 4F and G). Indeed, there was no significant difference in PAR stability between cells containing macroH2A1 (Luc KD cells) and macroH2A1-depleted (mH2A1 KD) cells with inhibited PARG (Fig. 4G). Overall, our data indicate that by binding to the ends of PAR chains, macroH2A1 alters PAR metabolism both by inhibiting PAR synthesis and by preventing PARG-mediated PAR degradation.

### MacroH2A1 facilitates repair of oxidative DNA damage in a pathway with PARP1.

**FIG 5:**
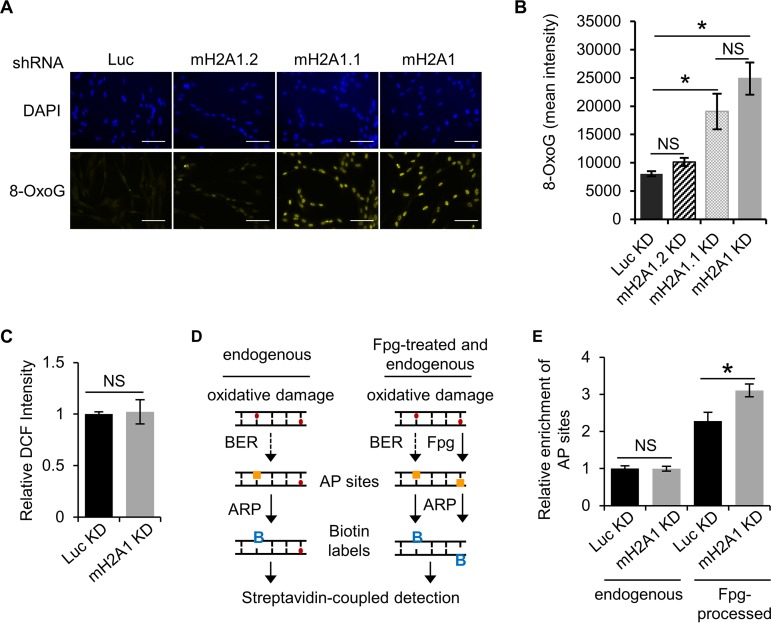
MacroH2A1 promotes repair of endogenous oxidative DNA damage. (A) Representative immunofluorescence images for 8-oxoG staining of IMR90 cells expressing shRNA against luciferase (Luc KD) as a control, both macroH2A1 isoforms (mH2A1 KD), macroH2A1.1 (mH2A1.1), or macroH2A1.2 (mH2A1.2). Scale bars, 100 μm. (B) Average mean intensities of 8-oxoG for three independent experiments as described in the legend to panel A. The bars and error bars represent means ± SEM. *, *P* < 0.05; Student's *t* test. NS, no significant difference. (C) ROS levels as measured by DCF fluorescence. The error bars show the standard errors of the mean across three biological replicates. (D) Schematic to detect oxidative DNA damage using an aldehyde-reactive probe (ARP) to biotin tag AP sites. Extracted genomic DNA was treated with Fpg to detect unprocessed sites of oxidative DNA damage. (E) Average relative enrichments of AP sites in Luc KD and mH2A KD IMR90 cells from DNA treated with Fpg or untreated. The error bars show the standard errors of the mean across three biological replicates. *, *P* < 0.05; Student's *t* test.

We have shown that macroH2A1 regulates the kinetics of PAR accumulation upon acute DNA damage. MacroH2A1 leads to a more sustained, stable accumulation of PAR while at the same time preserving cellular NAD^+^ levels by preventing PARP1 overactivation and preventing degradation of PAR chains. Overall, these activities allow macroH2A1.1 to prevent PARP-mediated necrosis in response to DNA damage. We next asked if the macroH2A1-dependent stabilization of PAR chains plays a role in DNA repair. We monitored 8-oxo-7,8-dihydroguanine (8-oxoG), as it is a major form of oxidative DNA damage and subject to PARP1-mediated BER (45–47). We observed a striking and significant increase in the endogenous level of 8-oxoG in both the macroH2A1-depleted and the macroH2A1.1-depleted cells relative to the control (Fig. 5A and B). Notably, there was no significant difference between control cells and cells depleted only of macroH2A1.2, indicating the increased 8-oxoG was primarily dependent on the absence of the PAR-binding macroH2A1.1 isoform.

Two distinct mechanisms may underlie the increased oxidative DNA damage seen in macroH2A1-depleted cells. Either macroH2A1 is involved in promoting efficient repair of these DNA lesions, or macroH2A1 reduces the formation of oxidative damage in the first place. Endogenous oxidative DNA damage is a consequence of cellular metabolism, which leads to the formation of reactive oxygen species (ROS) that react with DNA and proteins (48). Using the ROS-activated fluorophore 2′,7′-dichlorofluorescein (DCF), we determined that cellular ROS levels in macroH2A1-depleted cells were equivalent to those in controls (Fig. 5C). From this, we can conclude that macroH2A1 likely suppresses oxidative DNA damage by promoting its efficient repair.

8-oxoG, along with other types of oxidative base damage, is predominantly repaired by BER, which requires a DNA glycosylase to remove the damaged base. The cleavage of the N-glycosidic bond of the damaged base creates the apurinic/apyrimidinic (AP) site. Given the data discussed above, we hypothesized that macroH2A1 plays a role in BER, which is promoted by PARP1 activity. We used a DNA damage assay to specifically tag AP sites with biotin, which could then be quantified by colorimetric detection (49). Depletion of macroH2A1 did not alter the relative number of AP sites (Fig. 5D) in our cells, suggesting that macroH2A1’s role in this pathway lies upstream of AP site formation. AP sites are formed when DNA glycosylases recognize a specific base adduct and remove the damaged base, such as OGG1 in the case of 8-oxoG. Fpg (formamidopyrimidine [fapy]-DNA glycosylase) is a broad-specificity glycosylase that acts both as an N-glycosylase and an AP lyase, creating AP sites at several types of DNA lesions, including 8-oxoG (50). By treating genomic DNA (gDNA) with Fpg, we can assess oxidative DNA damage as the sum of AP sites and 8-oxoG. There was a significant increase of AP sites in Fpg-treated genomic DNA from macroH2A1-depleted cells (Fig. 5C), demonstrating that macroH2A1 likely promotes an early step in BER.

**FIG 6:**
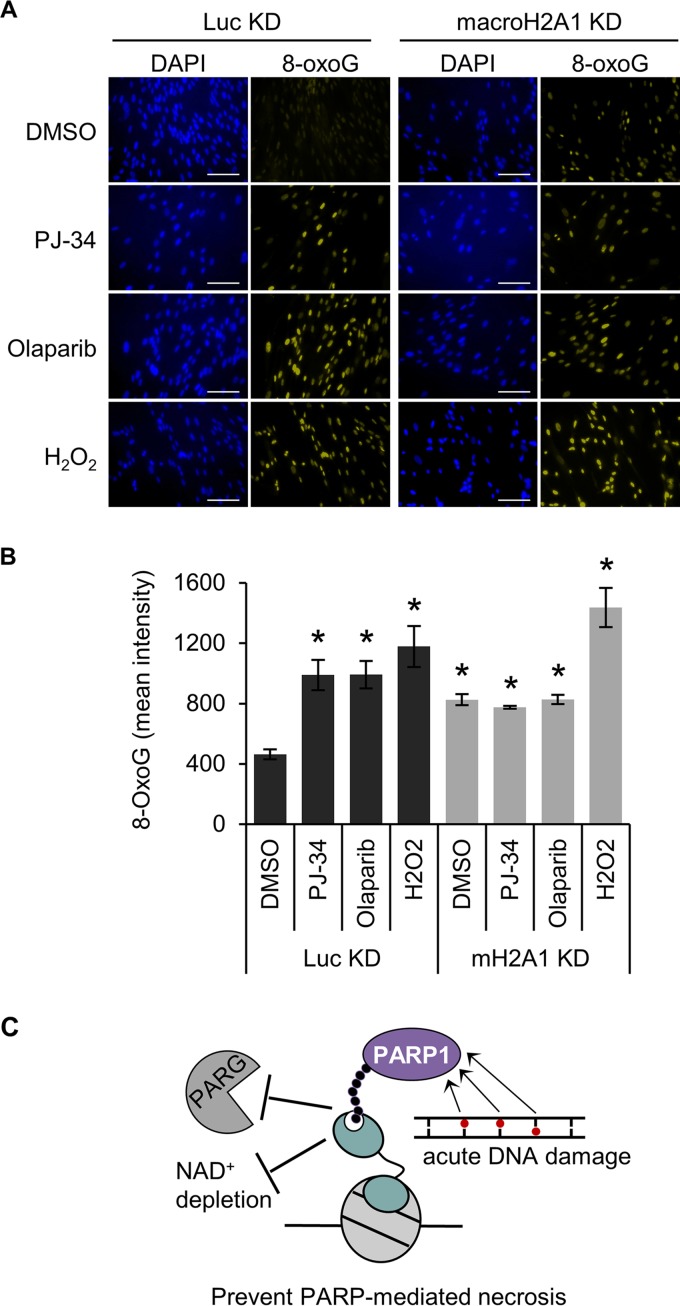
MacroH2A1 and PARP activities are epistatic for repair of oxidative DNA damage. (A) Representative immunofluorescence images for 8-oxoG staining of IMR90 cells expressing shRNA against macroH2A1 (mH2A1 KD) or luciferase (Luc KD) treated with DMSO, 10 μM PJ-34, or 1 μM olaparib for 3 days. Scale bars, 100 μm. (B) Average mean intensities of 8-oxoG for three independent experiments as described in the legend to panel A. The bars and error bars represent means ± SEM. *, *P* < 0.05 relative to Luc KD cells treated with DMSO; Student's *t* test. (C) Proposed model showing that macroH2A1.1-dependent stabilization of PAR chains by antagonizing PARG and inhibition of PARP1 activity prevents NAD^+^ depletion, prevents PARP-mediated necrosis, and increases the efficiency of PARP-mediated DNA repair.

The results discussed above suggest that macroH2A1 promotes the repair of damaged bases at the early step of base recognition and/or removal. Interestingly, previous work has shown that either decreasing PARP1 expression or chemical inhibition of PARP1 can impair BER, resulting in increased oxidative DNA damage (51, 52). In agreement with these findings, we showed that PARP1 inhibition by either olaparib or PJ-34, in the absence of H_2_O_2,_ increases steady-state 8-oxoG levels in cells containing macroH2A1 (Fig. 6). Notably, these PARP inhibitors function by distinct mechanisms, with olaparib trapping PARP1 on the damaged DNA (53) whereas PJ-34 competitively inhibits NAD^+^ binding (54). Still, both inhibitors led to increased oxidative DNA damage to the same extent in cells containing macroH2A1, suggesting that this result is not simply due to trapped, inactive PARP1 blocking access to repair intermediates, as has been suggested (55), and is instead due to the loss of PARP1 activity. Notably, PARP1 inhibition did not further increase oxidative DNA damage in cells depleted of macroH2A1, demonstrating that PARP1 and macroH2A1 are epistatic components of the BER pathway (Fig. 6). Altogether, these results indicate that macroH2A1 and PARP1 function in the same pathway to promote the repair of oxidative DNA damage.

## DISCUSSION

Histone variants and chromatin modifications play key roles in regulating DNA damage responses. For example, phosphorylation of H2AX triggers the recruitment of repair factors to sites of DNA double-strand breaks (56). In addition, a variety of modifications of canonical histones have been implicated in DNA repair (57). Our data expand the role of chromatin in regulating DNA damage responses by demonstrating that the functions of PARP1 and the histone variant macroH2A1 converge during DNA repair. PARP1 activity and subsequent PARylation are required for efficient recruitment of repair factors and to increase chromatin accessibility during the repair process. However, the PAR chains, which are critical to efficient DNA repair, are subject to degradation by glycohydrolases. MacroH2A1 plays a vital role in preventing PARP-mediated necrosis following acute DNA damage. MacroH2A1 both represses the rate of PAR synthesis and simultaneously represses the rate of PAR degradation to preserve cellular NAD^+^ levels, allowing PAR chains to accumulate in response to DNA damage while at the same time restricting PARP1 overactivation by capping PAR chains.

The macrodomain of macroH2A1.1 specifically interacts with the free ends of PAR chains (35). PARPs require access to the ends of PAR chains in order to continue polymerization. Therefore, macroH2A1.1 suppresses PARP1 activity by competing with PARP1 for access to the ends of PAR chains. Our data also indicate that macroH2A1 stabilizes PAR against the action of poly(ADP-ribose) exoglycohydrolases. While structural studies seem to suggest that ARH3 functions as an exohydrolase (58), there has been some controversy in the field regarding whether PARG functions predominantly as an exo- or endoglycohydrolase (9, 10, 59). Our finding that macroH2A1.1, a histone variant that binds the ends of PAR chains, can inhibit PAR turnover is strong evidence that in cells the dominant activities that degrade PAR are exohydrolytic in nature.

We demonstrated that by binding to the ends of PAR chains the macrodomain of macroH2A1 inhibits global PARP activity and thereby preserves cellular NAD^+^. PARP overactivation can promote cell death triggered either by the accumulation of PAR chains or by the depletion of NAD^+^. An essential cellular cofactor, NAD^+^ participates in redox reactions critical for ATP-generating processes, including glycolysis and oxidative phosphorylation. The depletion of cellular NAD^+^ that occurs during PARP overactivation leads directly to energy starvation through the inhibition of these pathways, followed by necrotic cell death (3). MacroH2A1.1 prevents this form of cell death simply by dampening PARP1 activity and, therefore, reducing NAD^+^ flux.

The PAR chains produced during excessive PARP1 activation by DNA damage can also directly participate in triggering cell death. PAR chains have been shown to directly inhibit hexokinase, an enzyme critical for glycolysis, which can lead to energetic catastrophe and cellular necrosis (60). PAR chains can also induce a form of programmed cell death, parthanatos, in which protein-free PAR chains are released from the nucleus, travel to the mitochondria, and initiate the translocation of AIF from mitochondria to the nucleus (61, 62). By interacting with the ends of PAR chains and protecting PAR from exoglycohydrolysis, macroH2A1.1 may give PARG time to cleave the chain by its less efficient endoglycohydrolytic mode, which could produce the protein-free PAR that is a prerequisite for parthanatos and hexokinase inhibition. Alternatively, macroH2A1.1 may help to anchor free PAR in the nucleus, thereby repressing these PAR-mediated cell death pathways. Importantly, we failed to observe changes in levels of extranuclear PAR during DNA damage responses in IMR90 cells, suggesting that in these cells parthanatos and hexokinase inhibition are not major mechanisms of PARP1-mediated cell death. In the future, it will be important to examine the effects of macroH2A1 on parthanatos and hexokinase inhibition in cell types and under conditions where these pathways are more prominent.

PARP1 can also function as a survival factor involved in DNA damage detection and repair. Damaged DNA allosterically activates PARP1, which then PARylates itself and other chromatin factors local to the damage, such as histone H1 and H2B (63, 64). Indeed, HPF1, a PARP-interacting factor, was recently shown to prevent PARP1 automodification and to promote PARylation of histones following DNA damage (65, 66). The PAR chains then aid in the recruitment of DNA repair factors to the sites of DNA damage (2, 67), including the endonucleases for both subpathways of BER (6, 68). Furthermore, ALC1, a PAR- and ATP-dependent chromatin remodeler, has been shown to be required to relax chromatin surrounding a DSB to mediate repair (69). A key objective in the future will be to determine how macroH2A1.1 affects this process. MacroH2A1 itself has previously been shown to be recruited to sites of DSBs and to promote DNA repair in its own right (29, 30, 70). One possibility is that by stabilizing PAR chains at sites of DNA damage, macroH2A1.1 helps promote the recruitment of repair factors. One previous study found that macroH2A1.1 promotes recruitment of 53BP1 to DSBs following ionizing radiation (29), providing supporting evidence for this hypothesis. In addition, the dependence on proximity of macroH2A1.1 in the genome to the site of DNA damage is also an important area for future investigation.

BER is initiated by DNA glycosylases, such as 8-oxoguanine glycosylase (OGG1), which recognize and remove the damaged base, producing a 3′ nick. The nicked DNA is recognized by an apurinic endonuclease, APE1, that promotes OGG1 disassociation and nicks the 5′ end of the sugar moiety to create a 1-base gap. This gap is then repaired by the SSBR machinery, which includes XRCC1, DNA polymerase β, and DNA ligase III (71). Interestingly, our data are consistent with an early role of macroH2A1, indicated by the fact that increased levels of 8-oxoG, but not increased numbers of AP sites, are found in the macroH2A1-depleted cells (Fig. 5). Another possibility is that macroH2A1-containing chromatin protects rather than repairs oxidative damage, as macroH2A1.2 has been demonstrated to localize to mediate protective chromatin compaction (70). Our data indicate a greater role for macroH2A1.1 in repair of oxidative lesions rather than protection, as the depletion of the macroH2A1.2 isoform did not increase 8-oxoG (Fig. 5B). Future studies are required to determine exactly how macroH2A1 contributes to 8-oxoG removal and if macroH2A1 potentially mediates other types of DNA repair.

Our data also indicate that PARP1 prevents 8-oxoG accumulation in a manner epistatic to macroH2A1 (Fig. 6), also suggesting an early role in BER. This is upstream of PARP1’s more established role promoting SSBR following recruitment and activation by the AP site (72, 73), where it generates a PAR scaffold to facilitate the association of the repair factors XRCC1, DNA polymerase β, and DNA ligase III (74–76).

There has been controversy regarding the requirement for PARP1 during BER (7). Several reports indicate that PARP1 promotes BER (45–47, 75, 77–81), whereas other studies indicate that PARP1 is not required or even inhibits BER (55, 82–85). The collaboration of macroH2A1.1 and PARP1 in BER described in this study may underlie these distinct results. Our data clearly indicate that macroH2A1 and PARP1 function together in a pathway that leads to the removal of 8-oxoG, an early step in BER. Observing the role of PARP1 in this process may require the presence of macroH2A1.1, the isoform that can interact with PAR chains. Importantly, macroH2A1.1 expression is often lost in cancer cells and transformed cell lines, explaining some of the disparate results regarding PARP1’s role in BER seen in the field.

As described above, counterintuitively, PARP1 can play two antagonistic roles in response to DNA damage. In essence, PARP1 functions as a rheostat; depending on the amount of damage, PARP1 can promote either repair and survival or cell death. Here, we show that macroH2A1.1 contributes to the set point of the PARP1 DNA damage rheostat by suppressing PARP1 overactivation and thereby dampening its ability to contribute to cell death pathways. This may have important implications for the use of PARP inhibitors in cancer treatment. In tumors harboring defects in homologous recombination, PARP1 function is required for cellular survival, and PARP inhibitors are showing promise as effective components of breast and ovarian cancer therapies (86, 87). The levels of macroH2A1.1 and macroH2A1.2 are often perturbed in many cancers (20–23, 88). While in lung and colon cancer macroH2A1.1 loss is a marker of poor prognosis (22, 23), it is interesting that the opposite association exists for triple-negative breast cancer, where macroH2A1.1 expression is a marker of poor prognosis (21). We hypothesize that in tumors with reduced levels of macroH2A1.1, PARP1 may be primed to function as a cell death effector in response to DNA-damaging agents, while in tumors that maintain normal levels of macroH2A1.1, PARP1 may function largely as a prosurvival factor. By monitoring macroH2A1.1 levels in patient biopsy specimens, we may be able to better target PARP inhibitors to those tumors where PARP1 plays a key role in tumor cell survival.

## MATERIALS AND METHODS

### Cell lines.

IMR90 primary human lung fibroblasts (ATCC), previously hTERT immortalized (20), were cultured in minimal essential medium (MEM) supplemented with 10% fetal bovine serum (FBS). Cell lines depleted of macroH2A1 or luciferase as a control were generated by retrovirus-mediated expression of shRNA using the pSuper.Retro system (OligoEngine). The targeting sequence for macroH2A1 was 5′- GCGTGTGTTGTGGTGCTTTAT-3′. The targeting sequence for macroH2A1.1 was 5′- GGCGACAAACACTGACTTCTA-3′. The targeting sequence for macroH2A1.2 was 5′- CTGAACCTTATTCACAGTGAA-3′. The luciferase shRNA targeting sequence was 5′-GATATGGGCTGAATACAAA-3′. The cells were selected and maintained under 0.5 mg/ml G418 in MEM supplemented with 10% FBS.

### Immunoblots and acid extraction of histones.

Cells were grown to 90% confluence in 10-cm dishes. The cells were lysed in 100 μl of detergent lysis buffer (10 mM Tris [pH 7.9], 0.1% Triton X-100, 100 mM NaCl, 1 mM EDTA, 5% glycerol, 1 mM dithiothreitol [DTT], 1× protease inhibitor cocktail). After 30 min of incubation on ice, the whole-cell lysate was centrifuged at 14,000 rpm for 10 min at 4°C. The pellet was resuspended in 80 μl 0.5 M HCl at 4°C for 2 h with agitation to extract the histones. The sample was spun at 14,000 rpm for 10 min at 4°C. The supernatant was neutralized with 20 μl 2 M Tris base. The acid extracts were subjected to SDS-PAGE and immunoblotting with primary antibodies, including macroH2A1.1 (Cell Signaling; 4106), macroH2A1.2 (Cell Signaling; 4827), and H3 (Abcam; AB1791). The whole-cell lysates were immunoblotted with PAR (Enzo; ALX-804-220-R100), PARP1 (Abcam; ab6079), vinculin (Sigma; V9131), GAPDH (glyceraldehyde-3-phosphate dehydrogenase) (Cell Signaling; 2118 L), and NMNAT1 (Santa Cruz; sc-271557). Horseradish peroxidase (HRP)-conjugated goat anti-rabbit or anti-mouse secondary antibody (Jackson Laboratory) was used for detection. Immunoreactivity was determined using an ECL kit (Thermo, Super Signal West Pico) following the manufacturer’s protocol. All immunoblotting was repeated at least twice with independent biological samples.

### RNA purification and RT-qPCR.

mRNA levels were analyzed by RT-qPCR. RNA was isolated with TriPure (Roche) according to the manufacturer’s protocol. The RNA was reverse transcribed with Moloney murine leukemia virus (MMLV) reverse transcriptase (Invitrogen) and a dT18 primer (an 18-mer deoxythymidine oligonucleotide). cDNA, SYBR green PCR master mix, and forward and reverse primers were used in 45 cycles of amplification (95°C for 15 s and 60°C for 1 min) following 10 min incubation at 95°C with a LightCycler 480 (Roche). The efficiency-corrected threshold cycle (Δ*C_T_*) method was used to determine the relative levels of RNA (89). For transcription analysis, expression was normalized to the human gene encoding beta-actin (*ACTB*). Melting-curve analysis was performed to ensure specificity. Primer sequences are listed in Table 1.

**TABLE 1:** Primer sequences

Gene name	Sequence	
	Forward	Reverse
*ARH3*	ACGCAGGCGGGTTTATTTTG	CCCCACATCCCATGTCCAAG
*PARG*	GACGCAATCTCTTCCACACA	TGAGTCAGGATGGAGGGAGT
*NMNAT1*	TCCGAGAAGACTGAAGTGG	GTAGGCATCACCAACAGG
*NMNAT2*	AGCAGTGCCTTGGTCTTGTT	GGGAACCCACTCCCCTATTA
*NMNAT3*	GCGCACATCCAGGAAATAGT	GATGGGAGATTCTGCGATGT
*NAMPT*	ATGGCAAGGTGACAAAAAGC	TGATGTGCTGCTTCCAGTTC
*PARP1*	GTGTGGGAAGACCAAAGGAA	TTCAAGAGCTCCCATGTTCA
*PARP2*	AACTGGGTGGGAATCTTGAG	TCTTTAGGCGAGAGGCAAAG
*PARP3*	CAAACTGGGTAATCGGAAGC	AGGCAAAGTAGATGCCCTTG

### Flow cytometry analysis.

For annexin V-PI assays, cells were stained with annexin V-fluorescein isothiocyanate (FITC) and PI and evaluated for apoptosis/necrosis by flow cytometry according to the manufacturer’s protocol (BD Bioscience). H_2_O_2_ was diluted in phosphate-buffered saline (PBS), added to the medium at 200 μM, and immediately added to the cells for a 90-min treatment. The cells were then washed in PBS, and fresh medium was added. After 8 h, the cells were trypsinized and recombined with cells floating in the medium. The collected cells were washed with cold PBS, stained with annexin V-FITC and PI in binding buffer for 15 min at room temperature in the dark, and analyzed using Canto II (Becton, Dickinson Biosciences). Cell populations were determined using FlowJo software (Tree Star Software, San Carlos, CA). The quadrants were chosen using unstained and single-stained control samples.

To quantify the level of reactive oxygen species, cells were washed briefly with warm PBS and incubated with 5 μM 2′,7′-dichlorodihydrofluorescein diacetate (H_2_DCFDA) (Invitrogen; C400) in PBS for 20 min. The cells were then quickly washed and allowed 20 min of recovery in MEM supplemented with 10% FBS. Cells were harvested with trypsin, resuspended in 0.5% bovine serum albumin (BSA) in PBS, and analyzed using Canto II (Becton, Dickinson Biosciences). Cell populations were determined using FlowJo software.

### Cytotoxicity assay.

Cells were seeded in a 96-well plate at 8,000 cells per well. Where indicated, the cells were pretreated for 30 min with 10 μM PJ-34 (Enzo; ALX-270-289-M005) or 1 μM olaparib (LC Laboratories; O-9201). H_2_O_2_ was diluted in PBS, added to the medium, and immediately added to the cells for a 90-min treatment. After H_2_O_2_ treatment, the cells were washed with PBS, and fresh medium was added. After 6 h, viability was measured with the CytoTox-Glo cytotoxicity assay (Promega; G9291), which is a fluorescence-based assay that measures a protease released only by necrotic cells, according to the manufacturer’s instructions. Briefly, cells were incubated in the dark for 15 min in AAF-Glo reagent. Then, luminescence was measured to determine the number of dead cells. The cells were then lysed with lysis reagent containing digitonin and incubated for 15 min at room temperature, and luminescence was measured to determine the total number of cells.

### NAD^+^ measurement.

Cellular NAD^+^ levels were quantified by means of an enzymatic cycling procedure (90). Briefly, cells grown in a 10-cm plate were treated with 125 μM H_2_O_2_ for the indicated times. The cells were collected in cold PBS and centrifuged at 5,000 rpm for 10 min at 4°C. The pellet was resuspended in 100 μl of 1 N HClO_4_ and neutralized with 50 μl of 1 N KOH. After the addition of 150 μl of 100 mM bicine, pH 8, 100 μl of the cell extract was mixed with 50 μl of the bicine buffer containing 0.114 M bicine (pH 7.8), 0.57 M ethanol, 4.8 mM EDTA-Na_4_, 1 mg/ml BSA, 0.48 mM 3-(4,5-dimethylthiazol-2-yl)-2,5-diphenyltetrazolium, 1.9 mM phenazine ethosulfate, and 48 μg/ml alcohol dehydrogenase. The mixture was incubated at room temperature for 20 min, and then the *A*_590_ was measured. A standard curve was used to quantify total NAD^+^.

### Immunofluorescence.

For all fluorescence experiments, 5 × 10^4^ cells were seeded onto coverslips (Fisher) in 12-well plates 24 h prior to staining. Images were captured on an Olympus IX81 microscope using an Olympus LCPlanFl objective at ×20 magnification with a numerical aperture of 0.4 at 20°C, using IP Lab 4.0.8 acquisition software. All images were exported as 16-bit files. The images were quantified over four separate fields containing at least 100 cells, using Volocity image analysis software. ImageJ was utilized for setting equivalent thresholds to generate representative image panels.

For PAR kinetic assays, cells were treated with 125 μM H_2_O_2_ and incubated for the times indicated. When indicated, cells were treated with 0.1 μM PDD00017273 (Sigma-Aldrich) to inhibit PARG activity at the peak of PAR intensity (indicated as time zero in Fig. 4A). The cells were then washed with PBS and fixed in 4% paraformaldehyde (Electron Microscopy Sciences; 15710-S) containing 10 μM PJ-34 and 50 μM tannic acid for 15 min at room temperature. The cells were then washed twice with PBS and permeabilized with 0.2% Triton X-100 in PBS for 10 min. The cells were then incubated with an antibody against PAR (ALX-804-220-R100) diluted 1:1,000 in 1% calf serum in 0.01% Triton X-100–PBS overnight at 4°C. The next day, the cells were incubated in Alexa Fluor 568-mouse secondary antibody diluted 1:1,000 for 1 h, mounted on slides using ProLong Gold antifade reagent with DAPI (4′,6-diamidino-2-phenylindole) (Life Technologies; P36935), and cured for 24 h prior to imaging. Nuclear PAR intensity values were analyzed by Volocity using a DAPI masking protocol. Coverslips were fixed and washed as described above and stained with macroH2A1.1 antibody (Cell Signaling; 12455S; 1:500) overnight and with goat anti-rabbit IgG conjugated to Alexa Fluor 488 (Life Technologies; A-11034; 1:1,000) for 1 h to detect macroH2A1.1 levels.

For 8-oxoG, the plates were put on ice at the time of harvest, washed twice in ice-cold PBS, and fixed in 100% ice-cold methanol for 30 min at −20°C. After fixation, the coverslips were washed thrice in PBS at room temperature and permeabilized with 0.01% Triton X-100–PBS for 15 min at room temperature. The coverslips were washed twice with PBS and treated with 500 μg/ml RNase A (Sigma) in TEN buffer (10 mM Tris-HCl [pH 7.4], 1 mM EDTA [pH 7.6], 400 mM NaCl) for 60 min at 37°C. After two PBS washes, DNA was denatured in 2 M HCl (freshly prepared) for 10 min at room temperature, washed twice with PBS again, and incubated with 0.1 M sodium borate (pH 8.5) for an additional 7 min. The cells were blocked for 1 h at room temperature in 5% fetal calf serum (in 0.01% Triton X-100–PBS). The cells were then incubated with anti-8-oxoG (Abcam; ab62623; 1:1,250) diluted in PBS with 1% calf serum (in 0.01% Triton X-100–PBS) overnight at 4°C. Following washing, the cells were incubated with goat anti-mouse IgG conjugated to Alexa Fluor 594 (Life Technologies; A-11005; 1:1,000) at room temperature for 1 h, washed, and mounted onto slides using ProLong Gold antifade reagent with DAPI (Invitrogen; P36935).

### AP site colorimetric measurement.

Total genomic DNA was extracted from Luc KD and mH2A KD IMR90 cells using a DNA Extractor WB kit (Wako; code no. 291-50502), and genomic DNA was kept on ice during the process. Buffers contained 100 μM desferal to prevent additional DNA oxidation. For Fpg enrichment, 5 μg of gDNA was digested with recombinant Fpg (New England Biolabs [NEB]; M0240S) and purified by ethanol precipitation; 0.1 μg Fpg enzyme was used for 1 μg of genomic DNA in NEB buffer 1 and BSA for 1 h at 37°C. Colorimetric measurement of AP sites was performed using a commercial kit (Abcam; ab211154) following the manufacturer’s protocol. The optical density at 450 nm was normalized using the standard curve of defined damage sites, and the numbers of AP sites are presented relative to gDNA from Luc KD IMR90 cells. The genomic-DNA concentration was determined using a Denovix QFX and a DeNovix double-stranded DAN (dsDNA) high-sensitivity fluorescence assay kit to ensure equal loading of genomic DNA.

### Statistical analysis.

All cytotoxicity, flow cytometry, immunofluorescence, and NAD^+^ measurement experiments were repeated at least 3 times and are presented as means ± standard errors of the mean (SEM). A two-tailed Student *t* test was used to determine the significance of differences between samples indicated in the figures. Fifty percent inhibitory concentrations (IC_50_s) and reaction parameters of PAR formation and PAR degradation were calculated using Prism version 7 (GraphPad Software, La Jolla, CA).
